# Supplementary material for: Identification and characterization of a membrane receptor that binds to human STC1
Source: Life Sci Alliance. 2022 Jul 7;5(11):e202201497. doi: 10.26508/lsa.202201497 (PMC9263378; doi:10.26508/lsa.202201497)
Supplement: Supplementary file 1 [file LSA-2022-01497_TableS1.docx]

**Supplementary Table S1:** Summary Binding Response Data

| **Ligand** | **Mol wt (kDa)** | **Theoretical Rmax(RUs)** | **Model** | ***k*_a_(1/Ms)** | ***k*_d_(1/s)** | ***k*_a2_(1/Ms)** | ***k*_d2_(1/s)** | **K_D_(nM)** | **Rmax**  **(RU)** | **%Rmax** | **Chi^2^(RU^2^)** |
| --- | --- | --- | --- | --- | --- | --- | --- | --- | --- | --- | --- |
| **hSTC1** | 27 | 1159 | Single site | 3.38x10^4^ | 5.14x10^-4^ |  |  | 15.20 | 896 | 77.3 | 344 |
|  |  |  | Two-state | 1.07x10^5^ | 2.93x10^-2^ | 8.68x10^-3^ | 5.63x10^-4^ | 16.75 | 1178 | 101.6 | 204 |
| **hSTC2** | 31.5 | 1352 | Single site | 9.50x10^5^ | 1.44x10^-3^ |  |  | 1.51 | 35 | 2.6 | 7.69 |
|  |  |  | Two-state | 3.21x10^6^ | 1.69x10^-2^ | 2.09x10^-3^ | 5.31x10^-4^ | 1.07 | 49 | 3.6 | 4.09 |
| **hCREG** | 21.9 | 940 | Single site | 1.34x10^5^ | 2.88x10^-4^ |  |  | 2.15 | 713 | 75.8 | 18.7 |
|  |  |  | Two-state | 2.06x10^5^ | 5.27x10^-3^ | 8.96x10^-3^ | 5.88x10^-4^ | 1.58 | 750 | 79.8 | 9.84 |
