# Supplementary figures and images for: Identification and characterization of a membrane receptor that binds to human STC1
Source: Life Sci Alliance. 2022 Jul 7;5(11):e202201497. doi: 10.26508/lsa.202201497 (PMC9263378; doi:10.26508/lsa.202201497)

## Slide 1
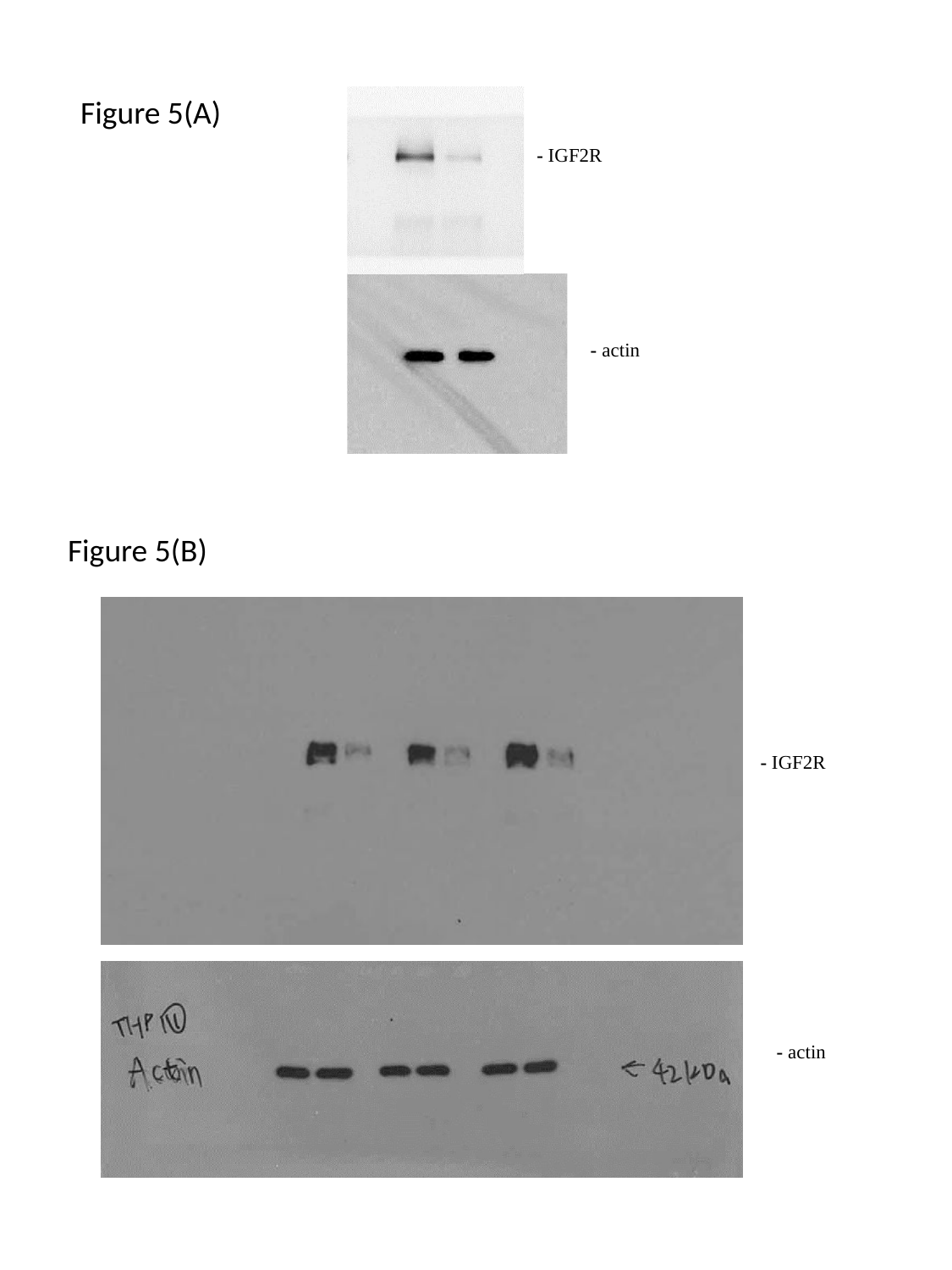

Figure 5(A)
- IGF2R
- actin
Figure 5(B)
- IGF2R
- actin

Supplement: Supplementary file 2 [file LSA-2022-01497_SdataF5.pptx]
